# Altered expression of anti-apoptotic protein Api5 affects breast tumorigenesis

**DOI:** 10.1186/s12885-023-10866-7

**Published:** 2023-04-25

**Authors:** Abhijith Kuttanamkuzhi, Debiprasad Panda, Radhika Malaviya, Gautami Gaidhani, Mayurika Lahiri

**Affiliations:** 1 Department of Biology, Indian Institute of Science Education and Research, Dr. Homi Bhabha Road, Pune, Maharashtra 411008 India; 2 The School of Chemistry and Molecular Biology, St. Lucia Campus, The University of Queensland, Brisbane, QLD 4072 Australia

**Keywords:** Breast cancer, Apoptosis, Api5, FGF2, Transformation, Proliferation, Morphogenesis

## Abstract

**Background:**

Apoptosis or programmed cell death plays a vital role in maintaining homeostasis and, therefore, is a tightly regulated process. Deregulation of apoptosis signalling can favour carcinogenesis. Apoptosis inhibitor 5 (Api5), an inhibitor of apoptosis, is upregulated in cancers. Interestingly, Api5 is shown to regulate both apoptosis and cell proliferation. To address the precise functional significance of Api5 in carcinogenesis here we investigate the role of Api5 in breast carcinogenesis.

**Methods:**

Initially, we carried out in silico analyses using TCGA and GENT2 datasets to understand expression pattern of API5 in breast cancer patients followed by investigating the protein expression in Indian breast cancer patient samples. To investigate the functional importance of Api5 in breast carcinogenesis, we utilised MCF10A 3D breast acinar cultures and spheroid cultures of malignant breast cells with altered Api5 expression. Various phenotypic and molecular changes induced by altered Api5 expression were studied using these 3D culture models. Furthermore, in vivo tumorigenicity studies were used to confirm the importance of Api5 in breast carcinogenesis.

**Results:**

*In-silico* analysis revealed elevated levels of Api5 transcript in breast cancer patients which correlated with poor prognosis. Overexpression of Api5 in non-tumorigenic breast acinar cultures resulted in increased proliferation and cells exhibited a partial EMT-like phenotype with higher migratory potential and disruption in cell polarity. Furthermore, during acini development, the influence of Api5 is mediated via the combined action of FGF2 activated PDK1-Akt/cMYC signalling and Ras-ERK pathways. Conversely, Api5 knock-down downregulated FGF2 signalling leading to reduced proliferation and diminished in vivo tumorigenic potential of the breast cancer cells.

**Conclusion:**

Taken together, our study identifies Api5 as a central player involved in regulating multiple events during breast carcinogenesis including proliferation, and apoptosis through deregulation of FGF2 signalling pathway.

**Supplementary Information:**

The online version contains supplementary material available at 10.1186/s12885-023-10866-7.

## Background

Apoptosis Inhibitor 5 (Api5), also known as Aac11 (Anti-apoptotic clone 11)/ MIG 8 (Migration Inducing Gene 8) and FIF (FGF2 interacting factor), is a 55 kDa protein localised in the nucleus [1]. The protein has a Leucine Zipper Domain (LZD), an LxxLL motif and a nuclear localisation signal (NLS) [2]. Tiwari et al*.* discovered Api5 as a cDNA clone that helped in the survival of cells upon serum deprivation [1]. Further studies identified its ability to regulate the expression of E2F1 target genes and thereby apoptosis and cell division [3, 4]. Api5 regulates apoptosis through caspase-2 inhibition and ERK2 mediated Bim degradation as well [5, 6]. A recent study from our lab has identified the regulators of Api5 acetylation and its’ requirement during the cell cycle [7].

Early reports started pointing at the possible tumour promoting role of Api5. Aac11 overexpression could protect human cervical cancer cells from apoptosis [8], while higher levels of API5 were associated with poor survival of NSCLC patients [9]. Api5 expression levels were observed to be elevated in Tamoxifen-resistant breast carcinomas [10]. Cho et al*.* reported higher expression of Api5 in cervical cancer tissues [11]. A recent report by Basset et al. shed more light on the role of Api5 in breast cancer, where they reported Api5 to interact with oestrogen receptor alpha (ERα) through the LxxLL motif. They also showed reduced in vivo tumorigenicity upon knock-down of Api5 in xenografted MCF7 cells in mice [12].

Interestingly, Di Benedetto and her group reported Api5 inhibition to reduce angiogenesis and increase in apoptosis in xenograft models, thereby highlighting the potential use of Api5 as a therapeutic target in metastatic breast cancers that are resistant to chemotherapy [13]. These reports suggested that Api5 may function as a tumour promoter by regulating various signalling mechanisms.

In our study, we report the role of Api5 in breast carcinogenesis using multiple models by altering the level of Api5 protein expression. Altered expression of Api5 affected proliferation, apoptosis, cell polarity and cell migration in both non- tumorigenic and tumorigenic cell lines. Api5 overexpression in MCF10A breast epithelial cells activated FGF2 signalling, leading to PDK1-Akt/cMYC activation during the early days of acinar morphogenesis. Activation of this led to elevated proliferation, migration, partial EMT and loss of polarity as was observed in Api5 overexpressed MCF10A cells. 3D cultures of these cells showed altered morphological changes and reduced apoptosis, which is speculated to be functioning through the FGF2-mediated activation of ERK signalling that was observed during the later days of acinar growth. Interestingly, reduced levels of Api5 resulted in decreased tumorigenic potential in the malignant breast cancer cell line, MCF10CA1a and impeded FGF2 signalling. Our findings provide insights into the importance of Api5 during several cellular signalling processes that, upon deregulation, can lead to breast carcinogenesis.

## Methods

### *In-silico* analyses

Using the GENT2 website, the subtype-based expression profile was plotted by selecting the subtype profile tab and providing “Api5” as the gene symbol. The survival plot is presented as provided by the tool with the overall survival of the patients. TCGA data (Legacy dataset) was downloaded from Xena Browser (UCSC Xena) by selecting IlluminaHiSeq dataset along with clinical data. Graph Pad Prism (Graph Pad Software, La Jolla, CA, USA) is used for plotting the extracted data. Further Kaplan Meier analysis was carried out using the kmplot online tool. Under breast cancer, “API5” was given as a gene symbol and “201687_s at” dataset was selected and using median cut-off, the survival plot was generated. Mutation data were analysed using the TCGA portal online tool. TCGA BRCA was selected as a dataset, and Api5 was given as a gene symbol.

### Cell lines and culture conditions

The MCF10A cell line was a generous gift from Prof. Raymond C. Stevens (The Scripps Research Institute, La Jolla, CA), while MCF10AT1 and MCF10CA1a were purchased from ATCC. These cells were grown as described earlier [1]. HEK 293 T cell line was a generous gift from Dr Jomon Joseph (National Centre for Cell Science, Pune, India). The cells were grown in DMEM with high glucose and sodium pyruvate (Invitrogen) containing 10% foetal bovine serum (Invitrogen), and 100 units/ml penicillin–streptomycin (Invitrogen). Stable cell lines overexpressing Api5 was prepared using lentiviral-mediated transduction. Briefly, HEK293T cells were transfected with 1 µg CSII-EF MCS mCherry Api5 vector having a mCherry-Api5 sequence, along with 0.5 µg pCMV-VSV-G-RSV-Rev and 1 µg pCAG-HIVgp for viral particle preparation using Lipofectamine 2000 (Invitrogen)-mediated transfection. Opti-MEM® used for transfection was obtained from Invitrogen. DMEM containing 15% horse serum was added to the cells 24 h post transfection. 48 h post transfection, viral supernatant was collected and filtered through a 0.45 µm filter to remove cell debris. The viruses were then used to transduce MCF10A. 4 µg polybrene was added to the cells to increase the transduction efficiency. As control, a stable cell line expressing only mCherry was also prepared. The transduced cells were sorted using BD FACS Aria (BD Biosciences) to get a pure population with maximum number of transduced cells. Api5 KD stable cells were generated in MCF10AT1 and MCF10CA1a in a similar manner. The shRNA was cloned in pLKO.1-EGFP vector, and packaging plasmids pMD2.G and pPax2 were used for lentiviral preparation. Cells were then sorted in BD FACS Aria.

### 3D ‘on-top’ culture

3D breast acinar cultures were set up in an 8-well chamber cover glass plates (Nunc Lab-Tek, Thermo Fisher Scientific) or 12-well plates (Eppendorf) using standard protocols [14, 15]. Cultures were grown in a humidified incubator with 5% CO2 and maintained at 37 °C (Eppendorf). The medium was changed every four days. For lysate collection on different days, higher cell density was seeded for Day 4 and Day 8.

For dissociation of acinar culture, Dispase™ (Corning, Sigma-Aldrich) was used. After the addition of Dispase™, the culture was incubated for 20 min. The dislodged acini were spun down at 900 rpm for 10 min, followed by 2 rounds of 1X PBS wash before plating in 12-well petri plates.

### Immunofluorescence staining

3D spheroid cultures were immune-stained with specific antibodies using established protocols [15]. For MCF10CA1a and MCF10AT1 spheroid staining, the 1X PBS and 1X IF buffer washes were added with 0.5% Triton X to aid for better penetration of antibodies. Images were captured using Leica SP8 or Zeiss LSM 710 laser scanning confocal microscope.

### Immunoblotting

Lysates from 2D or 3D cultures were collected in lysis buffer containing 50 mM Tris–HCl, pH 7.4, 0.1% Triton X-100, 5 mM EDTA, 250 mM NaCl, 50 mM NaF, 0.1 mM Na_3_VO_4_ and protease inhibitors. Immunoblotting against specific proteins was performed as per established protocols [16]. Images were acquired in ImageQuant LAS4000 (Cytiva, USA). The blot images represented shows all the bands that were captured using the imaging system. The entire blots were cut as per experimental requirements and probed for different antibodies as our model provides limited protein load and our blotting setup cannot carry out multiple antibody probing together. There are no images where two or more blots were merged.

### Immunohistochemistry

Formalin-fixed and paraffin-embedded breast cancer patient samples were collected from Prashanti Cancer Care Mission, Pune. Staining was carried out as per standard protocol [17]. The analysis was carried out by observing slides at 20X magnification of the compound microscope.

H-score for IHC is calculated as follows:

$$H-Score = Intensity\;score\;X\;Percentage\;positivity\;score$$

The intensity score and percentage positivity score ranges from 0 to 3, and the maximum H score is 9. Three individuals calculated the scores independently by observing ten different positions on the slide corresponding to the tissue ID. The median value from this observation was plotted on the graph.

### Single cell migration

Single cell migration analysis was performed as described earlier [18]. The data was processed using Fasttracks software [19] (FastTracks (https://www.mathworks.com/matlabcentral/fileexchange/66034-fasttracks), MATLAB Central File Exchange. Retrieved June 1, 2021) and parameters such as speed, displacement, distance, and persistence were calculated and plotted using Graph Pad Prism (Graph Pad Software, La Jolla, CA, USA).

### Soft agar assay

Soft agar colony formation assay was performed as detailed earlier [15]. Images were acquired using the 10X objective of a Nikon Eclipse TS-100 microscope. Ten randomly selected fields were imaged, and the colonies were manually counted.

### *In-vivo* tumorigenicity assay

6 × 10^6^ cells were injected subcutaneously in the flanks of athymic mice (Foxnl ^nu^ / Foxnl ^nu^, 6–8 weeks old) mixed with 1:1 diluted Matrigel® PBS mixture. Tumour size was measured with a vernier calliper. Furthermore, after 8–12 weeks, mice were sacrificed, and the tumour was dissected out. The tumour was then fixed with 10% formaldehyde and embedded in paraffin wax. The tumour area was calculated by multiplying the major and minor axis of the tumour as measured using a vernier calliper. The study is reported in accordance with the ARRIVE guidelines.

### Statistical analysis

Different morphometric parameters were tested for significance using the Mann–Whitney test. The significance test for the *in-silico* data was performed using either Mann–Whitney or Kruskal- Wallis test. The Mann–Whitney U-test was used to analyse the statistical significance of the relative Golgi area changes and the relative fluorescence intensity following immunostaining in the 3D cultures. The different parameters for analysing cell migration was tested for significance using the Mann–Whitney test. Statistical analysis for mice tumour area was performed using the 2-way ANOVA followed by Sidak’s multiple comparison test. *P* < 0.05 was considered statistically significant. Graph Pad Prism software (Graph Pad Software, La Jolla, CA, USA) was used to analyse data.

## Results

### Api5 transcript level is up-regulated in breast cancers and is associated with poor patient survival

**Fig. 1:**
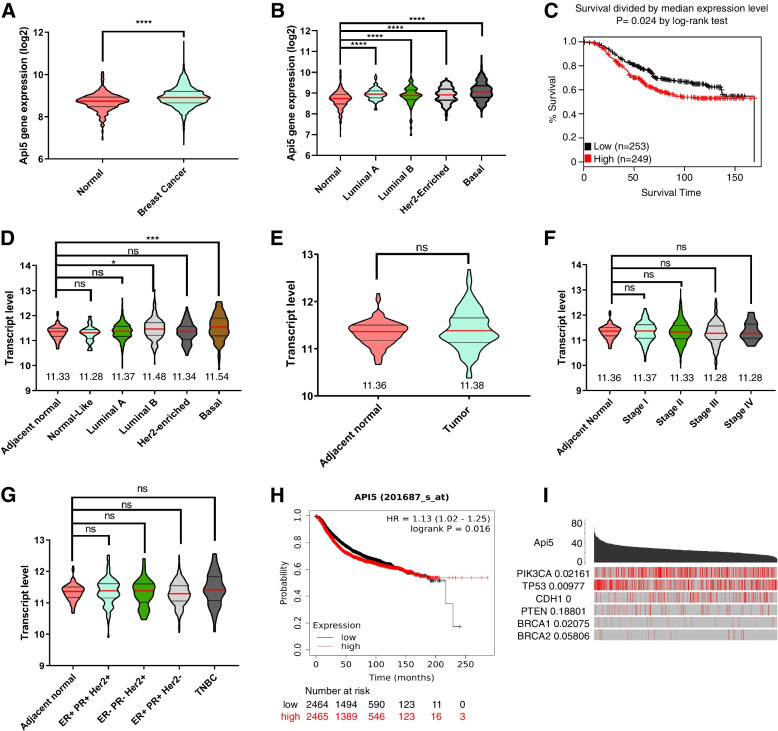
Api5 transcript levels are up-regulated in breast cancer and is associated with poor patient survival. **A** Transcript expression data (log2) of Api5 obtained from the GENT2 database is plotted. Significantly higher expression of Api5 is observed in cancer samples than in normal. Statistical analysis was performed using the Mann–Whitney test. **B** API5 expression data downloaded from GENT2 was used to compare the expression across different subtypes in comparison to the adjacent normal. Statistical analysis was performed using the Kruskal–Wallis test followed by Dunn's post hoc test. **C** Kaplan Meier plot showing survival probability of breast cancer patients divided into high or low API5 expression (Median cut-off). Blackline indicate the survival probability of low API5 expressing patients, and red indicates high API5 expression. Significance test data is provided by the online tool. **D** API5 transcript level expression obtained from TCGA database and manually analysed using Graph Pad Prism and plotted. Expression of API5 across different molecular subtypes was compared to the adjacent normal sample. Transcript expression of API5 from TCGA database compared across (**E**) adjacent normal and tumour tissues, (**F**) different stages of breast cancer, and (**G**) receptor status-based subtypes. Statistical analysis was performed using the Kruskal–Wallis test followed by Dunn's post hoc test. (The results published here are in whole or part based upon data generated by the TCGA Research Network: https://www.cancer.gov/tcga). **H** Kaplan Meier plot showing the probability of patient survival compared between API5 mRNA high or low samples. Data obtained from online tool kmplot. Expression data is divided based on the median value of the expression. Black points show low API5 expression while red shows high API5 expression. The significance test was carried out by the online tool and displayed as provided. **I** API5 expression and occurrence of mutations in genes were compared and plotted using the TCGA portal online tool. A significance test was performed using the online tool and displayed as provided. For all statistical tests, *P < 0.05, **P < 0.01, ***P < 0.001and ****P < 0.0001

To investigate the expression pattern of *API5* in breast cancers, in silico analysis was performed to compare *API5* expression between normal and tumour tissues of the breast using the GENT2 database [20]. The plot shows Api5 transcript levels are up-regulated in breast cancer tissue samples when compared to normal breast tissues (Fig. 1A and B). Further Kaplan–Meier survival analysis demonstrated poor survival of patients with high Api5 expression (Fig. 1C).

When similar analyses were performed using the TCGA dataset, the basal subtype showed higher expression of *API5* when compared to the other molecular subtypes and adjacent normal tissues (Fig. 1D-G).

Survival analysis in KM plotter [21] suggested that higher *API5* expression is associated with poor breast cancer patient survival (Fig. 1H). Using TCGA portal [22] online tool, we found higher Api5 expression is associated with mutations in PIK3CA (PI3-kinase catalytic subunit alpha), TP53 and CDH1 (E-cadherin), suggesting the possible deregulation of critical pathways associated with high API5 levels (Fig. 1I).

Furthermore, immunohistochemical analyses on breast cancer tissue samples showed that tumour tissues showed significantly higher Api5 expression when compared to the adjacent normal or reduction mammoplasty tissues. Also, Api5 expression levels were high in Stage 2 breast cancer samples in comparison to Stage 1 (Additional file 1A-D).

Elevated expression of Api5 in tumour tissues supports the possibility that Api5 may be a tumour promoter in breast malignancies. To investigate this further, we decided to overexpress Api5 in a non-tumorigenic breast epithelial cell line and study its effects on acinar phenotypes.

### Api5 overexpression in non-tumorigenic breast epithelial cells alters acinar morphogenesis due to an increase in proliferation

**Fig. 2:**
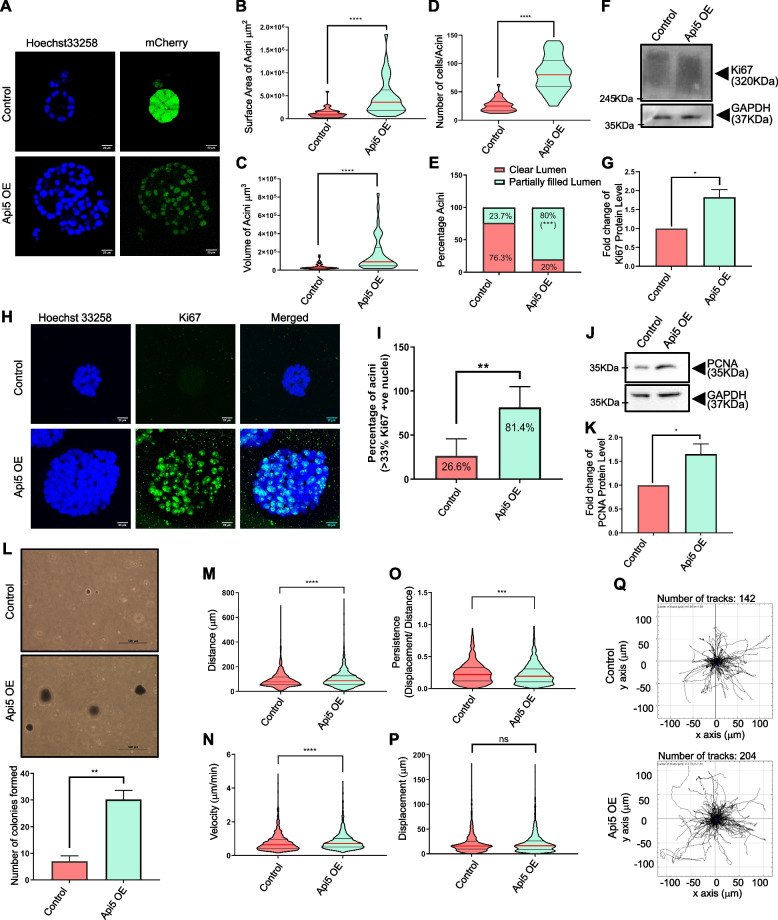
Overexpression of Api5 in MCF10A cells alter acinar morphology with increased proliferation. **A** Representative image of day 16 acini showing nuclei stained with Hoechst 33258 (blue) and mCherry (green). Violin plot showing (**B**) surface area and (**C**) volume of day 16 acini measured using Huygens software (SVI, Hilversum, Netherlands). **D** Violin plot showing the number of cells in each acinus, manually counted in day 16 Hoechst 33258-stained acini. Statistical analysis was performed using the Mann–Whitney test. **E** Bar diagram showing the percentage of acini with partially or entirely filled lumen. Day 16 acini stained with Hoechst 33258 were analysed manually by Huygens software. **F** Lysates collected from Api5 OE day 16 acini were immunoblotted for Ki67. **G** Quantification showing fold change in Ki67 protein level normalised to GAPDH (Samples from same experiment processed in 2 parallel gels as GAPDH is not detectable on 6% gel due to lower molecular weight). Statistical analysis was performed using the paired t-test. **H** Representative image of day 16 acini immunostained for Ki67 (green) and nuclei with Hoechst 33258 (blue). **I** The percentage of acini with more than 33% Ki67 positive nuclei were manually analysed and plotted as a bar graph. Statistical analysis was performed using an unpaired t-test. **J** Protein expression of PCNA in day 16 acinar cultures was obtained using western blot and quantified (**K**). Statistical analysis was performed using the paired t-test. **L** Representative image showing colony formed on soft agar assay and represented as a bar graph showing the total number of colonies formed. Statistical analysis was performed using the Mann–Whitney test. Control and Api5 OE cells were sparsely seeded and tracked for 3 h to analyse (**M**) distance travelled (**N**) velocity, (**O**) persistence and (**P**) displacement. **Q** Representative data showing movement of single cells in control and Api5 OE grown as monolayer cultures, sparsely seeded and tracked for 3 h. Statistical analysis was performed using the Mann–Whitney test. Data was pooled from N ≥ 3 independent experiments. For all statistical tests, *P < 0.05, **P < 0.01, ***P < 0.001and ****P < 0.0001. Full

To study the effect of Api5 overexpression on the cellular transformation of the breast acini, Api5 was overexpressed in MCF10A cells (will be called Api5 OE henceforth) (Additional file 2A). Following 16 days of culturing, it was observed that Api5 OE acini (Fig. 2A) have a larger surface area (Fig. 2B), volume (Fig. 2C), increase in the number of cells per acini (Fig. 2D) and filled lumen phenotype (Fig. 2E). There was no difference in the sphericity of Api5 OE acini when compared to the control acini (Additional file 2B). Similarly, Api5 overexpression did not produce protrusion-like structures in the 3D cultures (Additional file 2C). Api5 OE acini were not growth-arrested by day 16 and continued to proliferate as increased Ki67 protein expression was observed when compared to the control (Fig. 2F-G). More than 80% of Api5 OE acini showed greater than six Ki67 positive nuclei per acini (6 cells per acini = approx. 33% of cell/acini) as shown in Fig. 2H-I. On checking for the expression of PCNA, another proliferation marker, in the Api5 OE acini, an increase in PCNA levels was also observed in Api5 OE when compared to the control (Fig. 2J-K), thus further confirming that Api5 overexpression leads to increased proliferation. To investigate whether Api5 OE resulted in cellular transformation, anchorage-independent growth of Api5 OE cells dissociated from the 16-day acini was investigated. Api5 OE cells formed colonies on soft agar (Fig. 2L), indicating that overexpression of Api5 leads to the transformation of non-tumorigenic breast epithelial cells grown as acinar cultures.

The transformation of epithelial cells can also lead to increased migratory potential. When single-cell migration analysis was carried out, a significant increase in the distance travelled (Fig. 2M), velocity (Fig. 2N), decrease in persistence (Fig. 2O) while no change in displacement (Fig. 2P) was observed in 3D dissociated Api5 OE cells when compared to the controls. The track data was further used for plotting the cellular movement on an XY plot using ImageJ (Fig. 2Q). The tracking parameters suggest that Api5 OE cells move faster and cover a longer distance than the control. Further Api5 OE cells showed lower persistence suggesting that the cells were moving in a zigzag fashion rather than taking a relatively straight direction as was observed in the control cells. To understand the transformative potential of Api5, Api5 OE MCF10A cells dissociated from the acinar cultures were injected subcutaneously into the flanks of athymic mice and followed for eight weeks to check their in vivo tumorigenic potential. The Api5 OE cells did not form tumours (data not shown) suggesting that overexpression of Api5 is capable of partially transforming breast epithelial cells grown as acinar cultures with diminished tumorigenic potential.

### Overexpression of Api5 in breast epithelial cells results in polarity disruption

**Fig. 3:**
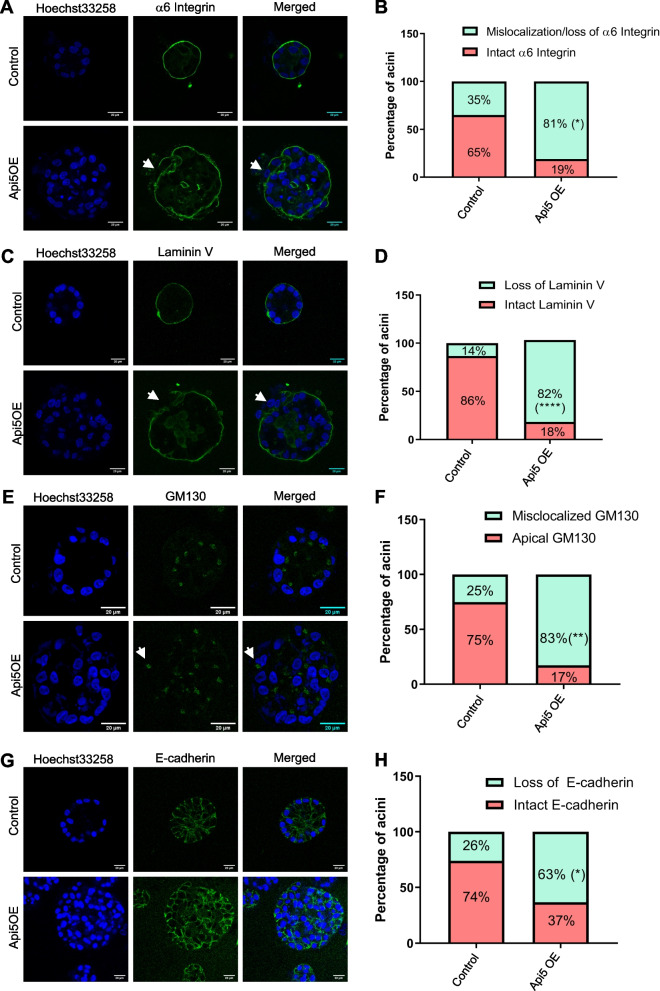
Overexpression of Api5 disrupts breast acinar polarity. **A** Representative image showing basal polarity marker, α6-integrin (green) immunostained in day 16 acini. **B** Bar diagram showing the percentage of acini with mislocalisation / loss of α6-integrin staining. **C** Representative image showing basal polarity marker Laminin V immunostaining (green) in day 16 control and Api5 OE acini. **D** Bar diagram showing the percentage of acini with mislocalisation/ loss of Laminin V staining. **E** Representative image showing GM130 (green) immunostaining in day 16 control and Api5 OE acini. **F** Quantification showing percentage of acini with mislocalised GM130 staining. **G** Representative image showing cell–cell junction marker E-cadherin (green) immunostaining in control and Api5 OE day 16 acini. **H** Bar diagram showing the percentage of acini with loss of E-cadherin staining. Statistical analysis for the percentage of acini was performed using an unpaired t-test. *P < 0.05, **P < 0.01, ***P < 0.001and ****P < 0.0001. Data pooled from N ≥ 3 independent experiments

Further, we studied the effect of Api5 OE on polarity in 3D acinar cultures. Api5 OE cells were cultured for 16 days and immunostained for the different polarity markers. α6-integrin and Laminin V, which marks the basal region of the acini [14] were observed to be mis-localised in around 80% of the Api5 OE acini compared to the control (Fig. 3A - D). Similarly, 83% of the Api5 OE acini had mis-localised GM130 (Fig. 3E and F), where GM130 was not observed to be apically positioned to the cell nucleus [14]. In addition, 63% of the Api5 OE acini showed loss of E-cadherin at cell–cell junctions (Fig. 3G and H). Interestingly, β-catenin, another cell–cell junction marker remained unaffected in the Api5 overexpressed acinar cultures (Additional file 2D and E). Taken together, our data suggest that overexpression of Api5 leads to polarity disruption in the breast acinar cultures, which can be associated with oncogene-mediated transformation. These results indicate that Api5 overexpression results in several characteristic changes in the epithelial cell line, possibly through epithelial to mesenchymal transition (EMT).

### Overexpression of Api5 in non-tumorigenic breast epithelial cells induce partial EMT-like characteristics

**Fig. 4:**
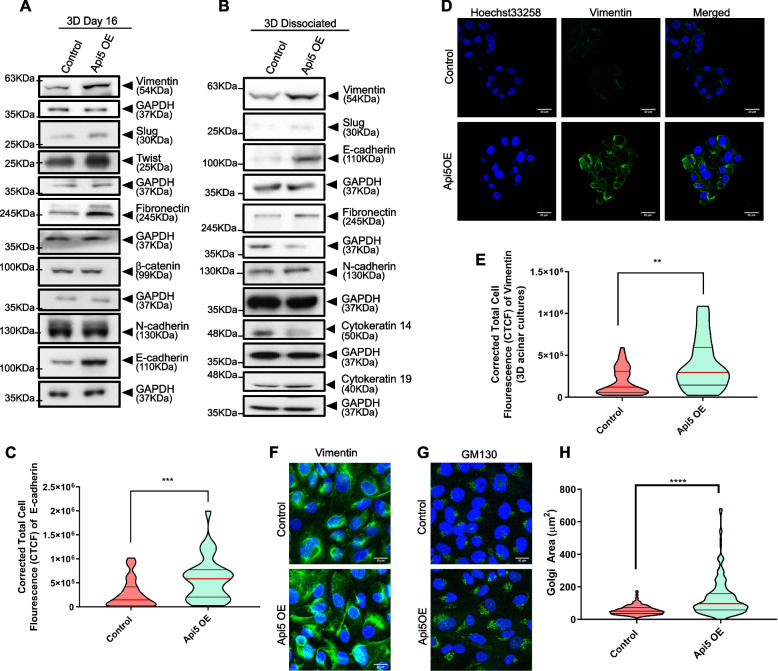
Api5 overexpression in breast acini leads to the epithelial cells acquiring partial EMT-like characteristics. Immunoblotting of lysates collected from (**A**) day 16 acini and (**B**) dissociated cells from day 16 acini and grown as monolayer cultures showing differential expression of epithelial and mesenchymal markers in Api5 OE compared to the control. **C** Violin plot showing corrected cell fluorescence of E-cadherin immunostaining of day 16 control and Api5 OE acini. **D** Representative image of Vimentin (green) immunostaining of control and Api5 OE day 16 acini. Nuclei were counterstained with Hoechst 33258 (blue). **E** Corrected cell fluorescence of vimentin quantified using ImageJ and represented as a violin plot. **F** Representative image of immunostained control and Api5 dissociated cells for Vimentin (green). Nuclei were stained with Hoechst 33258 (blue). **G** Representative image of GM130 immunostained control and Api5 OE dissociated cells (GM130: green, nuclei: blue). **H** Golgi area was measured using ImageJ. Statistical analysis was performed using the Mann–Whitney test. *P < 0.05, **P < 0.01, ***P < 0.001and ****P < 0.0001. Data pooled from *n* > 5 independent experiments

To study whether overexpression of Api5 leads to EMT, Api5 OE cells were cultured for 16 days, lysates collected, and immunoblotted. Vimentin, twist, slug and fibronectin showed 1.5-fold upregulation in Api5 OE acini when compared to the control (Fig. 4A and Additional file 3A-D). However, there was no change in β-catenin and N-cadherin expression levels (Fig. 4A and Additional file 3E and F). Interestingly the epithelial marker, E-cadherin showed a twofold increase in Api5 OE cells in comparison to the control (Fig. 4A, and Additional file 3G). This was confirmed by calculating the corrected fluorescence intensity of E-cadherin in day 16 acini (Fig. 4C) where a significant increase in the fluorescence intensity was observed, thereby suggesting an increase in E-cadherin levels. To further investigate whether the EMT-like phenotype that was observed upon overexpression of Api5 was a transient phenomenon or not, cells dissociated from the 16-day breast acinar cultures were grown as monolayer cultures and analysed. Similar to the phenotypes observed in the acinar cultures, an increase in vimentin, slug, fibronectin and E-cadherin were observed in the Api5 OE dissociated cells (Fig. 4B and Additional file 3H-K). N-cadherin expression remained unaltered in the Api5 OE dissociated cells (Fig. 4B and Additional file 3L). Both the epithelial cell markers cytokeratin 14 and 19 showed differential regulation in the Api5 OE dissociated cells. Cytokeratin 14 was downregulated while cytokeratin 19 was up-regulated (Fig. 4B and Additional file 3 M and N). The upregulation of vimentin was further confirmed when the 16-day Api5 OE breast acini, as well as the dissociated cells, were immunostained for vimentin and increased fluorescence intensity was demonstrated for the mesenchymal marker (Fig. 4D-F and Additional file 3O). Thus, taken together our data suggests that overexpression of Api5 leads to a partial EMT-like phenotype in the breast epithelial cells.

Glandular epithelial structures like breast acini have intact Golgi localised towards the apical region while some transformed or cancerous cells have dispersed Golgi [23]. Api5 OE cells were dissociated from 3D cultures and immunostained for the cis-Golgi marker GM130. It was observed that Api5 OE dissociated cells showed a significant increase in Golgi area when compared to the control, thereby suggesting an aberrant Golgi phenotype (Fig. 4G and H). Api5 OE in MCF10A cells grown as acinar cultures resulted in significant characteristic changes in the epithelial cell line thus resulting in cellular transformation.

### Reduced expression of Api5 in premalignant and malignant breast cancer cells led to partial reversal of cancerous phenotypes

**Fig. 5:**
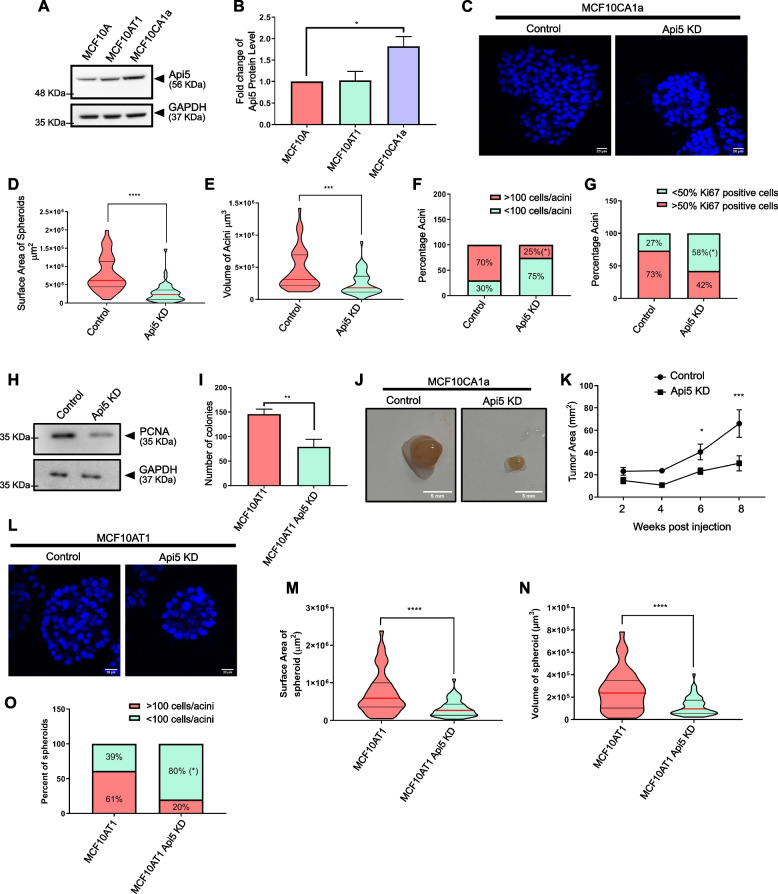
Api5 knock-down in malignant breast cells resulted in a partial reversal of cancerous phenotypes. **A** Api5 protein expression in MCF10A cell line series. **B** Quantification showing the expression levels of Api5 across the MCF10A cell line series normalised to loading control GAPDH. Statistical analysis was performed using the paired t-test. **C** Api5 KD and control MCF10CA1a cells cultured on Matrigel® for seven days stained with nuclear stain Hoechst 33258 (blue). MCF10CA1a control and MCF10CA1a Api5 KD spheroids were stained with phalloidin, and morphometric analysis was performed to calculate (**D**) surface area and (**E**) volume of spheroids using Huygens software (SVI, Hilversum, Netherlands). Statistical analysis was performed using the Mann–Whitney test. **F** The percentage of cells with > 100 or < 100 cells per spheroids were calculated and plotted. **G** Percentage of spheroids with more than 50% Ki67 positive cells per spheroid. Statistical analysis was performed using an unpaired t-test. **H** Immunoblotting of lysates collected from day 7 3D culture probed for PCNA. **I** Plot showing the total number colonies formed on soft agar. Statistical analysis was performed using the Mann–Whitney test. **J** Representative image of tumour dissected from flanks of athymic mice, 8 weeks post subcutaneous injection. The left panel shows MCF10CA1a control cells, while the right panel shows Api5 KD MCF10CA1a (Number of mice:—6). **K** Graph showing tumour area as measured using vernier calliper at the mentioned time. Statistical analysis was performed using 2-way ANOVA followed by Sidak’s multiple comparisons test. Data are pooled from *n* > 5 independent experiments. **L** Api5 KD and control MCF10AT1 cells were cultured on Matrigel.® for 7 days and stained with Hoechst 33258. Morphometric analysis was performed on MCF10AT1 control and Api5 KD spheroids stained with phalloidin and (**M**) surface area and (**N**) volume of spheroid were measured using Huygens software. Statistical analysis was performed using the Mann–Whitney test. **O** The number of cells per spheroids was counted based on nuclear staining and plotted for the percentage of spheroids with > 100 (red) and < 100 cells (green). Statistical analysis was performed using an unpaired t-test. Data pooled from N ≥ 3 independent experiments. For all statistical tests, *P < 0.05, **P < 0.01, ***P < 0.001and ****P < 0.0001

Since overexpression of Api5 was leading to a transformed phenotype, we wanted to explore whether down-regulation of Api5 could alter the cancerous phenotype in pre-malignant and malignant breast cancer cells. On investigating the protein expression of Api5 in the MCF10 cell line series [24–26], Api5 protein expression was observed to be up-regulated in the malignant MCF10CA1a cells in comparison to the non-tumorigenic MCF10A or pre-malignant MCF10AT1 cells (Fig. 5A-B). shApi5 knock-down stable cells were prepared in both MCF10AT1 and MCF10CA1a (Additional file 4A and G). When Api5 KD MCF10CA1a cells were cultured for 8 days, they formed smaller spheroids as was observed by a significant reduction in the surface area and volume when compared to MCF10CA1a control (Fig. 5C-E). This reduction in size was further corroborated with a significant reduction in the number of cells forming the Api5 KD MCF10CA1a spheroids (Fig. 5C and F). Interestingly, 58% of Api5 KD MCF10CA1a spheroids showed a reduction in proliferation as was analysed where less than 50% cells per spheroid were Ki67 positive (Fig. 5G and Additional file 4B). The levels of PCNA was also reduced upon knockdown of Api5 (Fig. 5H and Additional file 4C). Furthermore, Api5 KD MCF10CA1a cells formed fewer colonies on soft agar thereby indicating that a reduction in Api5 expression negatively affected anchorage-independent growth (Fig. 5I and Additional file 4D). When these Api5 KD MCF10CA1a cells were injected into the flanks of athymic mice they formed tumours, although the tumour volume was significantly reduced compared to the control MCF10CA1a cells. It was observed that while the MCF10CA1a control tumours continued to grow, Api5 KD MCF10CA1a tumours stopped growing and maintained a size similar to that of week 2 tumours (Fig. 5J and K, Additional file 4E and F).

Similarly, knock-down of Api5 in the pre-malignant cell line MCF10AT1 also led to a reduction in the surface area and volume of the spheroids (Fig. 5L-N). 80% of spheroids formed by Api5 KD MCF10AT1 had less than 100 cells per spheroid (Fig. 5O). Interestingly, knockdown of Api5 in MCF10AT1 spheroids did not affect proliferation as there was similar Ki67 expression in control and Api5 KD cells (Additional file 4H-I). Similar to the results observed in Api5 KD MCF10CA1a cells, knock-down of Api5 in the MCF10AT1 cells also resulted in a reduced ability to grow on soft agar suggesting a partial reversal of the malignant phenotype (Additional file 4 J-K).

### Api5 regulates FGF2-mediated Akt and ERK signalling

**Fig. 6:**
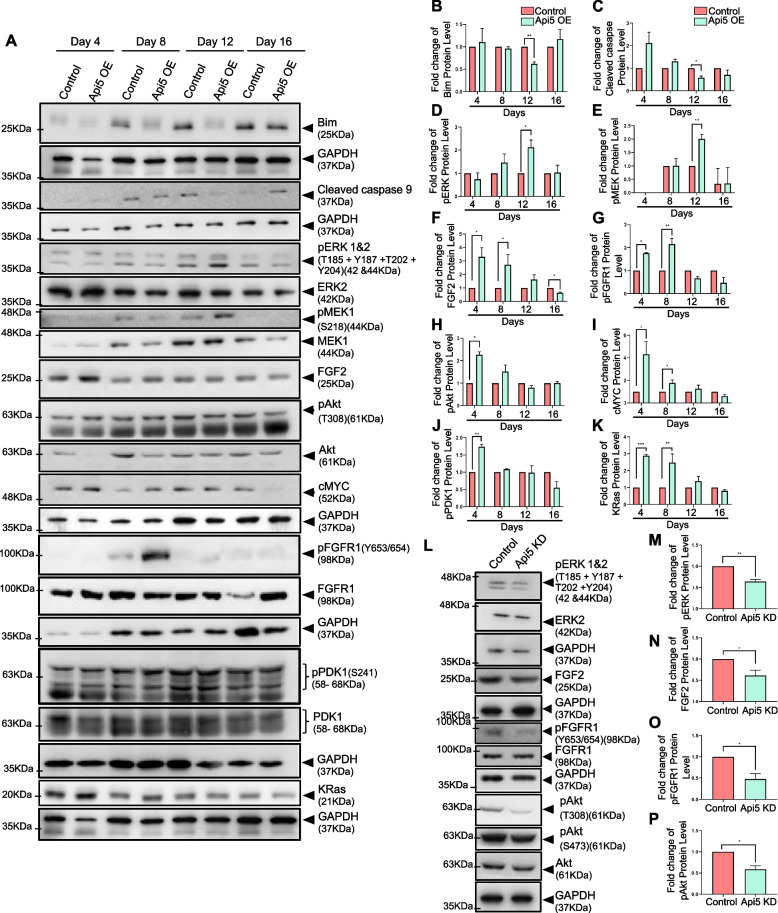
Api5 regulates PDK1/Akt and ERK pathways through FGF2. **A** Lysates were collected from control and Api5 OE acinar cultures on days 4, 8, 12 and 16 and immunoblotted to study the expression of a number of proteins. Fold change in expression between control and Api5 OE were calculated for (**B**) Bim, (**C**) cleaved caspase 9, (**D**) pERK 1/2, (**E**) pMEK1, (**F**) FGF2, (**G**) pFGFR1 (Y653/654) (**H**) pAkt (T308), (**I**) cMYC, (**J**) pPDK1 (S241) and (**K**) KRas. The normalisation of expression was done with GAPDH for Bim, cleaved caspase 9, cMYC, and FGF2, while for pERK, pMEK, pFGFR1, pPDK1 and pAkt, it was with their respective total protein. (For pFGFR1 and FGFR1, samples from same experiment processed in 2 parallel gels as probing with pFGFR1 antibody affects binding of FGFR1 antibody). **L** Immunoblotting of various proteins involved in the plausible molecular pathway using lysates collected from day 7 MCF10CA1a control and Api5 KD spheroid cultures. Quantification of the fold change in expression of (**M**) pERK 1&2 normalised to total ERK2, (**N**) FGF2 normalised to GAPDH, (**O**) pFGFR1 normalised to total FGFR1, and (**P**) pAkt T308 normalised to total Akt. Data pooled from *n* > 5 independent experiments. Statistical analysis was performed using the paired t-test. *P < 0.05, **P < 0.01, ***P < 0.001and ****P < 0.0001. Data pooled from N ≥ 3 independent experiments. (Note: In 6A- pFGFR1 blot, day 4 samples are reprobed separately to overcome the masking effect, this data is added to Additional file 5B)

To further understand the importance of Api5 in breast carcinogenesis, it is essential to delineate the molecular signalling involved in the various processes. Initially, we investigated the effect of Api5 OE on apoptosis. Overexpression of Api5 led to a reduction in Bim, and active caspase 9 on day 12 of morphogenesis (Fig. 6A-C). Since ERK-mediated Bim degradation has been reported to be through Api5 signalling [5], we checked whether overexpression of Api5 could lead to an alteration in ERK and MEK activity. Phosphorylation of ERK and MEK kinases were observed upon Api5 OE during day 12 of acinar growth (Fig. 6A, D and E). Also, an increase in FGF2 protein expression was observed from days 4 to 12 in Api5 OE acini when compared to the control. This increase was a little over threefold on day 4 while it was 1.5-fold on day 12. By day 16, FGF2 was marginally lower in Api5 OE cells compared to the control (Fig. 6A and F). Earlier studies have reported API5-induced immune resistance to be dependent on FGF2-mediated activation of FGFR1 receptor [5]. Similarly, in our study, we observed increased FGFR1 phosphorylation on days 4 and 8 that also coincided with higher FGF2 levels (Fig. 6A and G). Although an increase in FGF2 levels was observed on day 4, an increase in ERK signalling was observed only from day 12. FGF2 is known to activate tyrosine kinase receptors that then activate the PI3K signalling cascade [27]. Overexpression of Api5 increased phosphorylation of Akt at T308 on days 4 and 8, while the Akt S473 phosphorylation was similar to that of the control cells (Fig. 6A, H and Additional file 5A). Therefore, it can be inferred that PDK1, which phosphorylates Akt at T308, is activated, which may lead to further activation of its downstream signalling molecule, cMYC. Upon probing for cMYC in these lysates, a significant increase in cMYC expression was observed in days 4 and 8 Api5 OE lysates, which coincided with the phosphorylation of Akt at T308 residue (Fig. 6A and I). Further studies confirmed PDK1 to get activated on day 4 in Api5 overexpressing cells which coincided with both phosphorylation of Akt at T308 residue and cMYC upregulation (Fig. 6A and J). Thus, the FGF2-mediated activation of PDK1-Akt/cMYC signalling during the initial days of growth supports the increased proliferation, protein synthesis and transformation of MCF10A cells, while later activation of the ERK signalling cascade leads to reduced apoptosis and lumen filling. FGF2- PDK1/ ERK signalling is very often linked through RAS and a recent study has shown that Api5 interacts with KRAS [28], thus KRAS expression pattern was also studied. Interestingly, KRAS was up-regulated in Api5 OE 3D lysates from day 4 to day 12 and by day 16, KRAS levels were similar to that of the control (Fig. 6A and K). Taken together, we propose Api5 to activate FGF2-mediated Ras-ERK and Akt signalling cascades.

Furthermore, Api5 KD resulted in a decrease in ERK phosphorylation (Fig. 6L and M). Also, reduction in both FGF2 protein and mRNA levels was observed in Api5 KD cells when compared to control (Fig. 6L and N, Additional file 5B and C). Immunoblotting of lysates from 3D spheroid cultures showed decreased FGFR1 activation when Api5 is knocked down in MCF10CA1a cells (Fig. 6L and O). Akt activation was also perturbed upon Api5 KD, as was demonstrated by diminished Akt phosphorylation at both S473 and T308 sites (Fig. 6L and P).

To further investigate the existence of this regulation in breast cancer patients, co-expression analysis was performed using the TCGA database. Interestingly, *BAX* and *CASPASE-9* negatively correlated with API5 transcript levels, but BIM mRNA levels positively correlated with *API5* (Additional file 5G-I). We observed a similar trend in the protein expression levels in Api5 OE MCF10A lysates collected on day 16. ERK2, FGF2, PDK1, KRAS and cMYC transcript levels also showed a positive correlation with API5 transcript (Additional file 5 J-M), supporting the data obtained from the 3D acinar cultures. These data indicate that Api5 is regulating the FGF2-mediated PDK1 and ERK signalling in breast cancer and can possibly be used as a target for therapy.

## Discussion

**Fig. 7:**
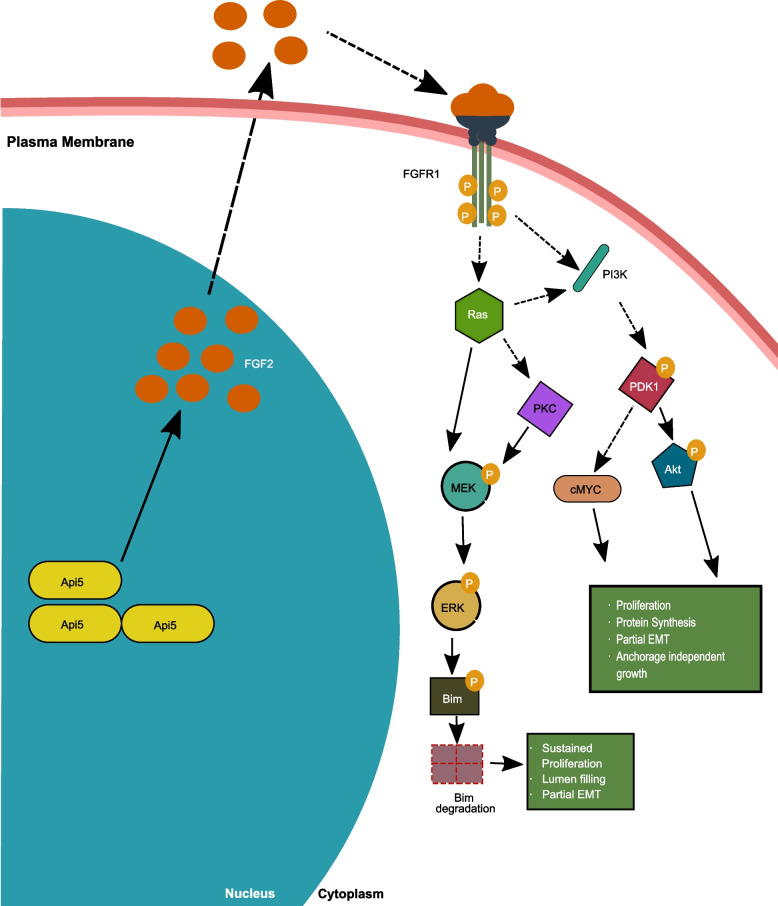
Schematic depicting the molecular mechanism of Api5-mediated transformation of breast epithelial cells. Api5 through FGF2 (High Molecular weight—nuclear localised) led to activation of FGFR1 growth factor receptor signaling. During the early days of morphogenesis, overexpression of Api5 led to elevated proliferation via the PDK1- Akt/cMYC pathway thus, aiding in the transformation of breast epithelial cells. cMYC is known to promote EMT and anchorage-independent growth was also observed in Api5 OE MCF10A cells. Further, during the later days of morphogenesis, FGF2 signalling activated ERK-mediated Bim degradation, thereby inhibiting apoptosis, and supporting sustained proliferation. Both the signalling cascades can contribute to the partial-EMT state observed in Api5 OE MCF10A cells. Thick lines show signalling mechanisms revealed from experiments conducted in this study while dotted lines represent signalling mechanisms obtained from published literature

Apoptosis is an important cellular process required during constant remodelling of glandular epithelium in the human breast, which is often deregulated in cancers [29]. The role of Api5, one of the regulators in the apoptotic signalling cascade, is not well established in breast carcinogenesis. In this study, we report that deregulation of Api5 expression in breast epithelial cells leads to activation of Akt and ERK signalling pathways that affect breast morphogenesis (Fig. 7). In this study, we report overexpression of Api5 results in the disruption of polarity, sustained proliferation, partial EMT-like characteristics and anchorage independent growth while the opposite trend was observed when Api5 was knocked down in malignant breast cells. Thus, Api5 may be playing a role in promoting breast tumorigenesis through its ability to regulate multiple cellular characteristics.

In order to understand the role of different genes in tumors, in silico analyses of patient derived genomic data is widely used by researchers. Analyses performed using multiple online tools and databases revealed that API5 transcript levels are higher in breast tumour tissues than in normal breast samples. A significant positive correlation was observed between *API5* expression and mutations in key signalling molecules such as p53 and PI3K. Mutations in these genes are known to be pathogenic and favour tumour progression [30, 31].

The human breast contains lobules made of numerous acini which produce milk. In each acinus, epithelial cells surround an empty lumen in which milk is secreted and is then carried through the ducts [32]. When cultured on specific extracellular matrices, breast epithelial cells can form similar structures in vitro [33]*.* MCF10A, a non-tumorigenic breast epithelial cell line, forms growth-arrested acinar structures when cultured on a laminin-rich extracellular matrix [14]. Overexpression of oncogenes such as Erbb2, Akt and cMYC led to morphometric changes in the MCF10A acini [34, 35]. Oncogenic transformation in 3D acinar cultures can result in a filled lumen, larger acinar size and formation of protrusions from the spherical structures [36]. These characteristic changes that epithelial cells acquire have been enumerated in the hallmarks of cancer, coined by Hanahan and Weinberg [37] such as resisting cell death, sustained proliferative signalling, invasion and metastasis.

In our study, MCF10A cells stably overexpressing Api5 were cultured as spheroids. We observed morphometric changes including an increase in size and cell number indicating the possibility of cellular transformation, thus suggesting a plausible role of Api5 in breast cancer. Furthermore, knock-down of Api5 in MCF10A isogenic cell lines, MCF10CA1a (malignant) [26] and MCF10AT1 (pre-malignant) [25] led to a reduction in both the size of spheroids as well as proliferation.

MCF10A acini have epithelial cell characteristics with basal and apical polarity [14]. Several reports have demonstrated that loss of polarity and reduced lumen size are associated with breast cancer progression [36, 38]. In our study, we observed a similar phenotype in MCF10A acini overexpressing Api5. Both basal markers (Laminin V and α6-integrin) and apical marker (GM130) were mis-localised at several regions in the Api5 OE MCF10A acini suggesting changes in cell characteristics.

Epithelial cell characteristics are mostly altered during cellular transformation [39]. Epithelial cells gain mesenchymal characteristics such as the expression of proteins like Slug, Twist, and Vimentin. We observed that Api5 OE led to a partial EMT-like phenotype. A partial/hybrid EMT state is observed when a cell expresses both epithelial and mesenchymal markers [40]. Recent reports have demonstrated that the hybrid EMT state helps cells to attain stemness characteristics and drug resistance [41], both of which are also associated with Api5-FGF2 signalling [42, 43]. Identifying whether Api5-induced partial EMT could drive drug resistance and induce stemness characteristics in cancer cells will aid in developing treatment strategies against chemo-resistant cancers.

Altered epithelial characteristics are known to affect the cell migration. Moreover, increase in migratory potential is a common phenotype observed in malignant cancer cells [44]. In some scenarios they follow collective cell migration while single cell migration is also observed. The single cells detached from the tumour travel to another tissue, often leading to metastasis [45]. We report that overexpression of Api5 led to increased single cell migration. The cells attained higher speed and travelled longer distance, however, with reduced persistence. A similar result was earlier reported when fibroblast and glioblastoma cells were treated with EGF [46]. A lower persistence indicates that the directionality of cellular movement is affected. Golgi plays a major role in cell migration and directionality of movement [47]. Since Api5 OE resulted in the dispersal of Golgi, this may be promoting the elevated migratory potential, however, with reduced persistence [48].

Further, we also demonstrated that Api5 regulates anchorage-independent growth, a well-established property suggesting malignant transformation. Api5 OE led to anchorage-independent growth of MCF10A cells, while knock down in MCF10CA1a and MCF10AT1 showed reduced colony formation on soft agar. When the Api5 KD cells (MCF10CA1a) were injected into the flanks of mice, they formed tumours, although the size was smaller when compared to MCF10CA1a control cells. However, Api5 OE cells did not form tumours in athymic mice. This suggests that overexpression of only Api5 may not be sufficient to cause complete transformation and carcinogenesis of breast epithelial cells. A series of deregulations and genomic changes are required for a cell to be completely transformed. Our results suggest that altered Api5 expression can affect various cellular characteristics and thus promote cellular transformation.

During MCF10A acinar morphogenesis, cells in the lumen undergo Bim-mediated apoptosis during days 10 to 16 [49]. Api5 OE led to reduced Bim levels, thereby inhibiting apoptosis. Studies have reported that oncogenes such as Erbb2 and v-Src show a similar inhibition of Bim, thus preventing luminal cell death [49]. This reduction in apoptosis explains the presence of a partially or entirely filled lumen that was observed in the Api5 OE acini. Bim protein levels are regulated by ERK signalling where ERK activation leads to phosphorylation of Bim and thereby its degradation [50]. We identified that Api5 OE activated FGF2-MEK-ERK signalling during day 12 of acinar morphogenesis, which coincided with the observed Bim degradation. Knock-down of Api5 in the malignant breast cell line confirmed that Api5 can regulate the FGF2-MEK-ERK signalling cascade (Fig. 7).

Api5 OE induced higher FGF2 expression from day 4 of acinar morphogenesis which activated PDK1-Akt/cMYC signalling. Knock-down of Api5 resulted in reduced activation of this signalling cascade. c-MYC expression is known to be up-regulated in several breast cancers. Higher expression is also predicted to cause poor patient outcome [51]. The elevated c-MYC expression also mediates EMT in breast cancers [52], thus possibly explaining the partial-EMT observed in Api5 OE cells. Interestingly, Partanen and group in 2007 reported that overexpression of c-MYC in MCF10A led to the filling of the lumen and possible transformation-like phenotypes, although interestingly, the organised and polarised acinar architecture suppressed the transforming capability of c-MYC [35]. Later Simpson et al*.* reported that endogenous and exogenous c-MYC expression is suppressed during later stages of MCF10A acinar cultures [53]. Interestingly, our results also indicate that Api5 overexpression induced altered signalling was restored by day 16 of acinar morphogenesis. Therefore, it is possible that acinar morphogenesis could have prevented a complete malignant transformation of overexpressed Api5 in MCF10A cells.

Our investigations have uncovered a detailed understanding of the role of Api5 in breast carcinogenesis. We report that Api5 through FGF2, regulates ERK and Akt signalling, thereby leading to the transformation of breast epithelial cells (Fig. 7). The ability of Api5 to regulate multiple signalling pathways may be an advantage for developing novel breast cancer treatment strategies. Our data indicate Api5 to be a major regulator of several key events during breast carcinogenesis including elevated proliferation, decreased apoptosis, polarity disruption and anchorage-independent growth. This study opens up a plethora of new research opportunities and new possibilities for drug development.

## Conclusion

Our study demonstrated the necessity of Api5 in breast carcinogenesis where we found the various cellular characteristics that Api5 can regulate. We have also identified a novel molecular signalling involved in Api5 mediated transformation of breast epithelial cells that functions through FGF2- KRas/PDK1-Akt/cMYC and FGF2- ERK-Bim pathways.

## Supplementary Information

**Additional file 1. **

**Additional file 2. **

**Additional file 3. **

**Additional file 4. **

**Additional file 5. **

**Additional file 6. **

**Additional file 7. **

**Additional file 8. **

**Additional file 9. **

**Additional file 10. **

**Additional file 11. **

## Data Availability

The datasets analysed during the current study are available in the TCGA and GENT2 repository. (GENT2: http://gent2.appex.kr/gent2/, TCGA BRCA dataset: https://portal.gdc.cancer.gov/projects/TCGA-BRCA).

## Abbreviations

API5: Apoptosis Inhibitor 5
FGF2: Fibroblast Growth Factor 2
TCGA: The Cancer Genome Atlas
GENT2: Gene Expression database of Normal and Tumor tissues 2
EMT: Epithelial—Mesenchymal Transition
OE: Overexpression
KD: Knockdown
PDK1: Phosphoinositide-dependent kinase-1
