# Supplementary figures and images for: Altered expression of anti-apoptotic protein Api5 affects breast tumorigenesis
Source: BMC Cancer. 2023 Apr 25;23:374. doi: 10.1186/s12885-023-10866-7 (PMC10127332; doi:10.1186/s12885-023-10866-7)

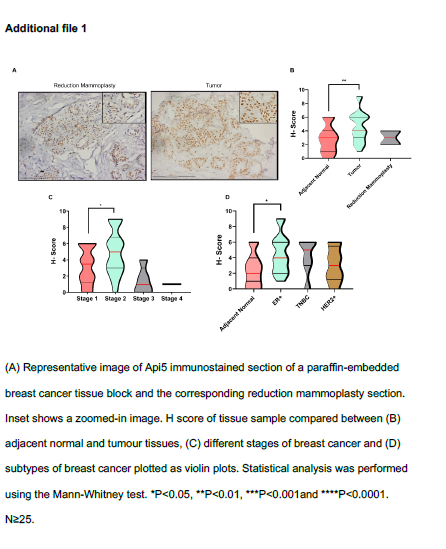

Supplement: Supplementary file 1 — Additional file 1. [file 12885_2023_10866_MOESM1_ESM.docx]

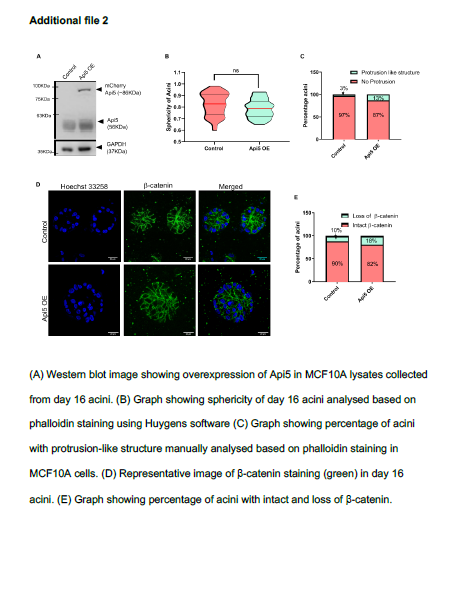

Supplement: Supplementary file 2 — Additional file 2. [file 12885_2023_10866_MOESM2_ESM.docx]

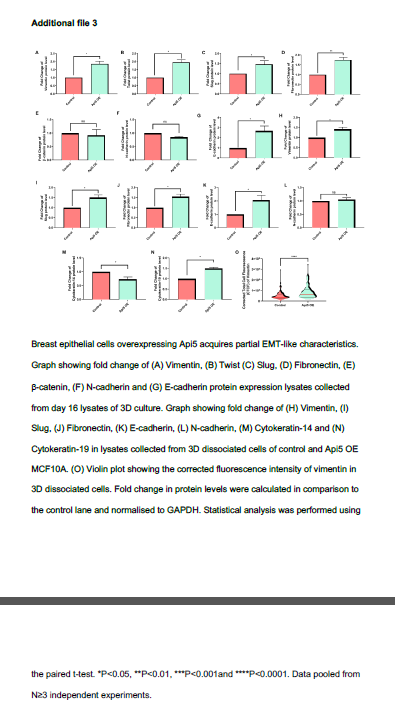

Supplement: Supplementary file 3 — Additional file 3. [file 12885_2023_10866_MOESM3_ESM.docx]

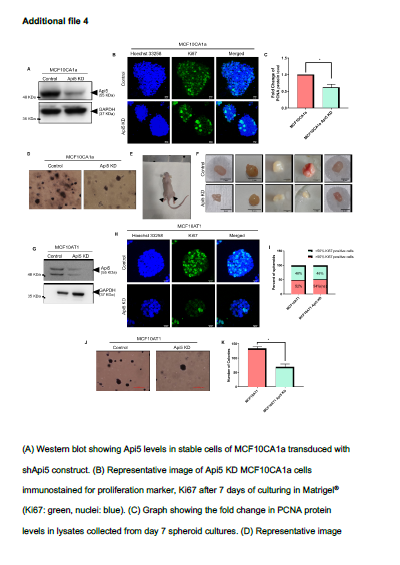


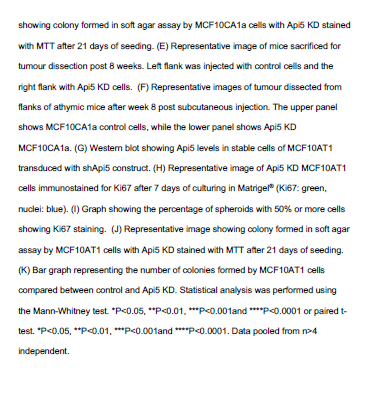

Supplement: Supplementary file 4 — Additional file 4. [file 12885_2023_10866_MOESM4_ESM.docx]

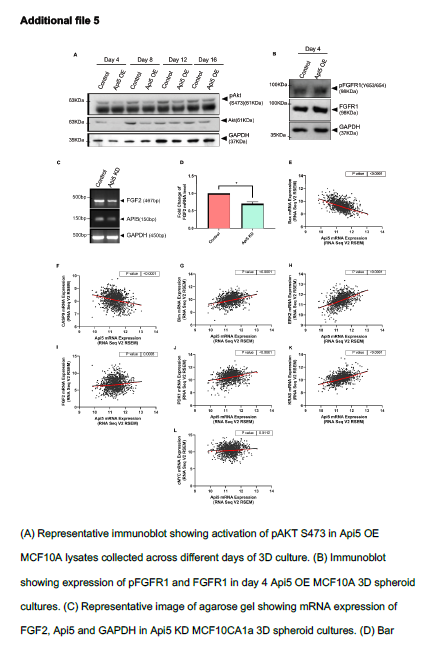


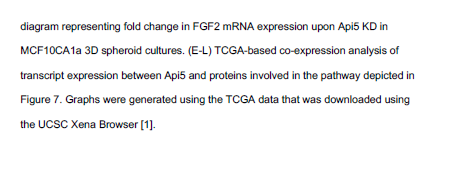

Supplement: Supplementary file 5 — Additional file 5. [file 12885_2023_10866_MOESM5_ESM.docx]

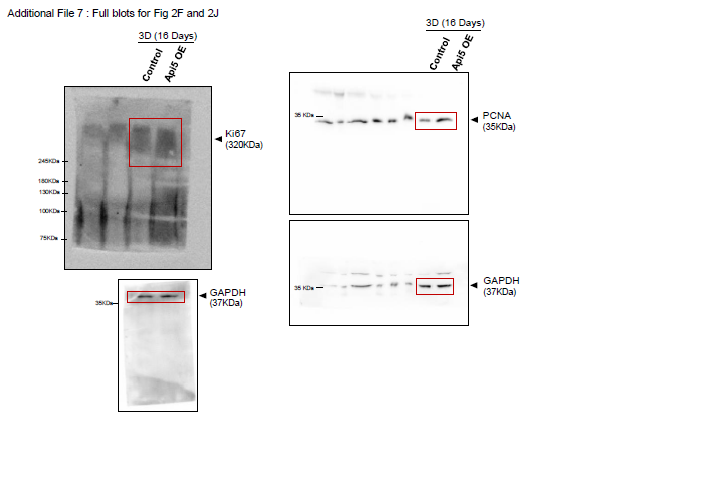

Supplement: Supplementary file 7 — Additional file 7. [file 12885_2023_10866_MOESM7_ESM.docx]

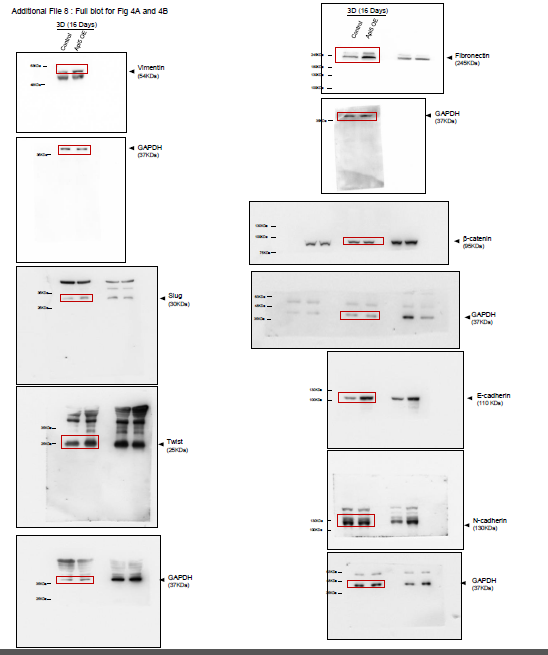


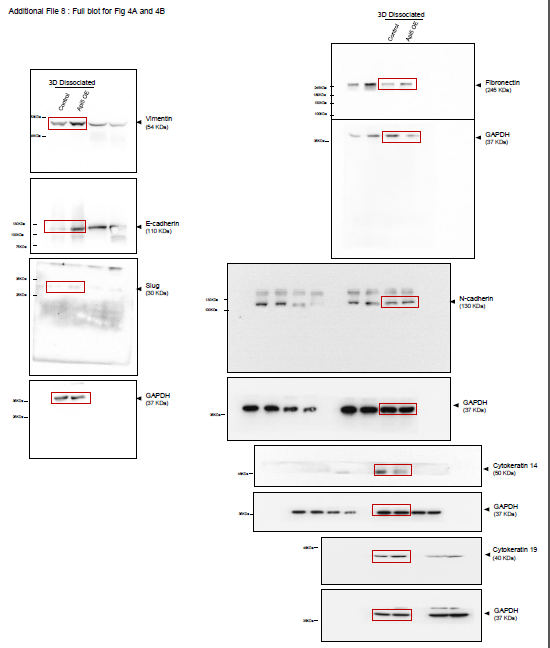

Supplement: Supplementary file 8 — Additional file 8. [file 12885_2023_10866_MOESM8_ESM.docx]

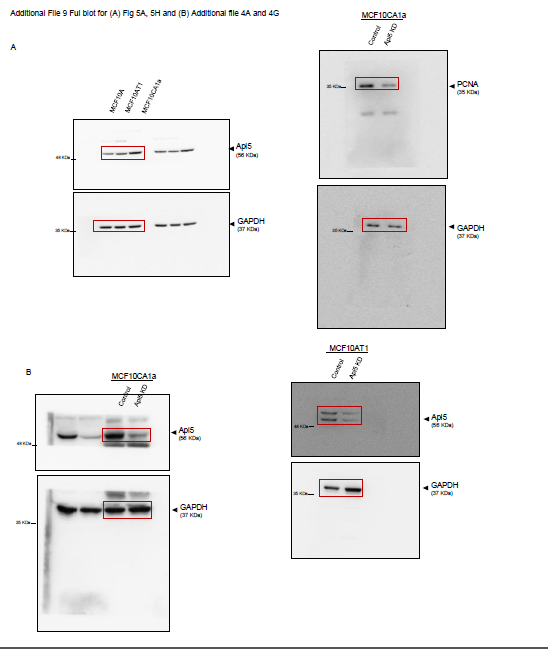

Supplement: Supplementary file 9 — Additional file 9. [file 12885_2023_10866_MOESM9_ESM.docx]

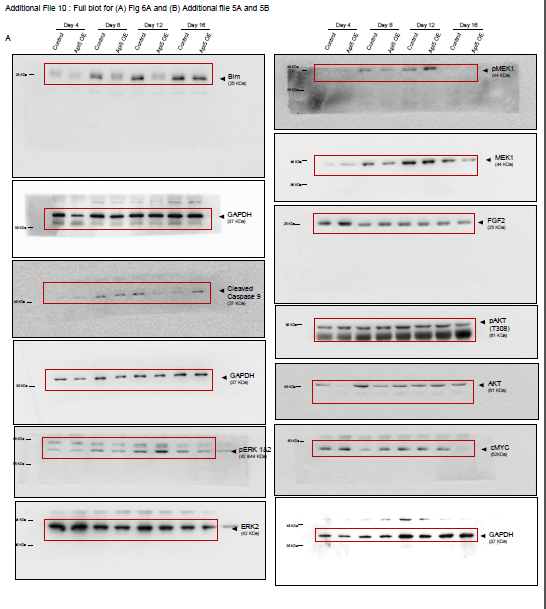


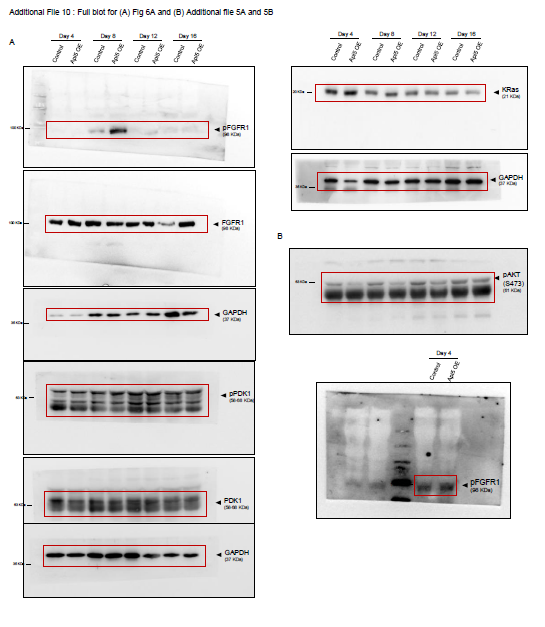

Supplement: Supplementary file 10 — Additional file 10. [file 12885_2023_10866_MOESM10_ESM.docx]

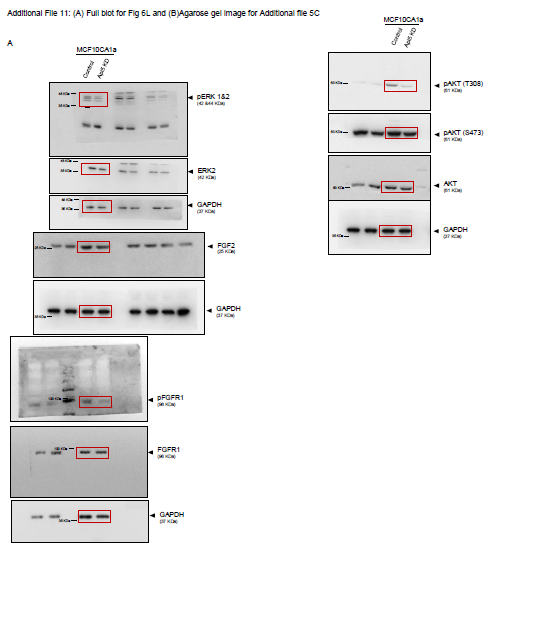


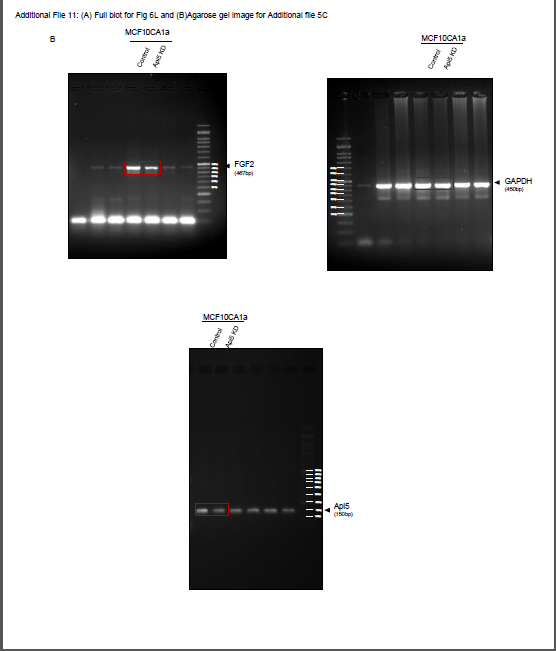

Supplement: Supplementary file 11 — Additional file 11. [file 12885_2023_10866_MOESM11_ESM.docx]
