# Supplementary material for: Altered expression of anti-apoptotic protein Api5 affects breast tumorigenesis
Source: BMC Cancer. 2023 Apr 25;23:374. doi: 10.1186/s12885-023-10866-7 (PMC10127332; doi:10.1186/s12885-023-10866-7)
Supplement: Supplementary file 6 — Additional file 6. [file 12885_2023_10866_MOESM6_ESM.docx]

**Additional file 6**

**Supplementary methods**

Chemicals and antibodies

Cholera Toxin (C8052), Epidermal Growth Factor (E9644), Hydrocortisone (H0888), Insulin (I1882), Polybrene (H9268), and Poly-L-Lysine (P8920) were purchased from Sigma-Aldrich. Lipofectamine-2000 (11668-500) was purchased from Invitrogen, Thermo Fisher Scientific. Dispase (354235) was purchased from Corning, Sigma-Aldrich. NaF (RM1081) Na2HPO4 (GRM1417), and KH2PO4 (MB050) were purchased from HiMedia. 16% paraformaldehyde (AA433689M) was purchased from Alfa Aesar.

Immunofluorescence staining was carried out using Ki67 (Abcam, monoclonal, ab16667), α6-integrin (Merck, monoclonal, MAB1378), Laminin V (Merck, monoclonal, MAB19562), GM130 (Abcam, polyclonal, ab30637), E-cadherin (Abcam, monoclonal, ab1416), Vimentin (Abcam, monoclonal, ab92547), and β-catenin (Abcam, monoclonal, ab32572). IHC against Api5 was performed using Api5 (Sigma polyclonal, HPA026598). Api5 (Sigma, polyclonal HPA026598 or Abnova, polyclonal, PAB7951), PCNA (Cell signalling, monoclonal, 2586), E-cadherin (BD, monoclonal, 610182), N-cadherin (Abcam, polyclonal, ab18203), GAPDH (Sigma, polyclonal, G9545), Vimentin (Abcam, monoclonal, ab92547), Slug (Cell Signalling, monoclonal, 9585), Twist (Abcam, Polyclonal, ab50581), Fibronectin (BD, monoclonal, 610077), β-catenin (BD, monoclonal, 610153), Cytokeratin 14 (Abcam, monoclonal, ab7800), Cytokeratin 19(Abcam, monoclonal, ab52625), Bim (Abcam, monoclonal, ab32158), Cleaved Caspase-9 (Abcam, polyclonal, ab2324), pERK 1&2 (Abcam, monoclonal, ab50011), ERK2 (Abcam, monoclonal, ab32081), pMEK1 (Abcam, monoclonal, ab32088), MEK1 (Abcam, monoclonal, ab32091), FGF2 (Millipore, monoclonal, 05-118), pAkt T308 (Cell Signalling, monoclonal, 4056), pAkt S473 (Invitrogen/ Biosource, monoclonal, 44-621G), Akt (Cell Signalling, monoclonal, 9272S), pFGFR1 Y653/654 (Cell Signalling, monoclonal, 3471S), FGFR1 (Cell Signalling, monoclonal, 9740S), pDPK1 S241(Cell Signalling, monoclonal, 3438S), PDK1 (Cell Signalling, monoclonal, 5662S) and cMYC (Santacruz, monoclonal, SC-40) were used for the immunoblotting experiments. Peroxidase-conjugated AffiniPure goat anti-mouse (115-035-003) and anti-rabbit(111-035-003), as well as AffiniPure F(ab′)2 fragment goat anti-mouse IgG, F(ab′)2 fragment specific (115-006-006), were obtained from Jackson Immuno Research. Hoechst 33342 (H3570), Hoechst 33258 (H3569), Alexa Fluor ® 488 conjugated anti-mouse secondary antibody(A-11029), Alexa Fluor ® 488 conjugated anti-rabbit secondary antibody (A-11034), Alexa Fluor ® 568 conjugated anti-mouse secondary antibody(A-11004), Alexa Fluor ® 568 conjugated anti-rabbit secondary antibody(A-11036), Alexa Fluor ® 568 conjugated anti-rat secondary antibody(A-11077), Alexa Fluor ® 633 conjugated anti-mouse secondary antibody (A-21052), Alexa Fluor ® 633 conjugated anti-rabbit secondary antibody (A-21071), and Alexa Fluor ® 568 phalloidin (A-12380) were bought from Invitrogen, Thermo Fisher Scientific.

Plasmids

CSII-EF-MCS plasmid was a gift from Dr Sourav Banerjee, NBRC, Manesar, India. pCAG-HIVgp and pCMV-VSV-G-RSV-Rev plasmids were purchased from RIKEN BioResource Centre. mVenusC1 was gifted by Jennifer Lippincott-Schwartz, NIH, USA in which Api5 was cloned.

Primers used for Api5 CDS insertion to CSII-EF-MCS vector were: Forward primer: 5’- AAGGAAAAAAGCGGCCGCATATGCCGACAGTAGAGGAGCT- 3’ and reverse primer: 5’-GCTCTAGATCAGTAGAGTCTTCCCCGAC - 3’.

pMD2.G and pPax2 were generous gift from Dr Manas Kumar Santra, NCCS, Pune, India. pLKO1.EGFP was a generous gift from Dr Sorab Dalal, ACTREC, Mumbai, India.

Primers used for shApi5 insertion to pLKO1 vector were: Forward primer: 5’- CCGGAAGACCTAGAACAGACCTTCACTCGAGTGAAGGTCTGTTCTAGGTCTTTTTTTG - 3’ and reverse primer: 5’- AATTCAAAAAAAGACCTAGAACAGACCTTCACTCGAGTGAAGGTCTGTTCTAGGTCTT - 3’.

Semi-quantitative PCR

RNA extraction and cDNA synthesis of Api5 KD MCF10CA1a 3D spheroids were performed as described previously [2]. Semi-quantitative PCR was performed using FGF2 forward (5′-ATGGCAGCCGGGAGCATCACCACG-3′), FGF2 reverse (5′-TCAGCTCTTAGCAGACATTGGAAG-3′), API5 specific forward (5′-CGAGTGGCAGATATACTAACGC-3′) and reverse (5′-TCCTCTCCTTGAAGTATTTGGC-3′) primers. GAPDH was used as endogenous control and was amplified using forward (5′-ACCACAGTCCATGCCATCAC-3′) and reverse (5′-TCCACACCCTGTTGCTGTA-3′) primers. The following PCR cycle was used for the amplification: 95 °C for 60 s, 58 °C (API5), 60°C (GAPDH) and 65°C (FGF2) for 45 s, 72°C for 60 s and final extension for 3 min.

1. Debnath, J., Muthuswamy, S. K. & Brugge, J. S. (2003) Morphogenesis and oncogenesis of MCF-10A mammary epithelial acini grown in three-dimensional basement membrane cultures, *Methods.* **30**, 256-68.

2. Anandi, V. L., Ashiq, K. A., Nitheesh, K. & Lahiri, M. (2016) Platelet-activating factor promotes motility in breast cancer cells and disrupts non-transformed breast acinar structures, *Oncol Rep.* **35**, 179-88.
